# Extracellular Matrix and Cytokines: A Functional Unit

**DOI:** 10.1155/2000/31748

**Published:** 2000

**Authors:** Elke Schönherr, Heinz-JüRgen Hausser

**Affiliations:** Institute of Physiological Chemistry and Pathobiochemistry University of Münster Waldeyerstrasse 15 Münster D-48149 Germany

**Keywords:** proteoglycan, glycosaminoglycan, TGF-β, FGF-2

## Abstract

The extracellular matrix (ECM) as well as soluble mediators like cytokines can influence the behavior of cells in very distinct as well as cooperative ways. One group of ECM molecules which shows an especially broad cooperativety with cytokines and growth factors are the proteoglycans. Proteoglycans can interact with their core proteins as well as their glycosaminoglycan chains with cytokines. These interactions can modify the binding of cytokines to their cell surface receptors or they can lead to the storage of the soluble factors in the matrix. Proteoglycans themselves may even have cytokine activity. In this review we describe different proteoglycans and their interactions and relationships with cytokines and we discuss in more detail the extracellular regulation of the activity of transforming growth factor-β (TGF-β) by proteoglycans and other ECM molecules. In the third part the interaction of heparan sulfate chains with fibroblast growth factor-2 (FGF-2, basic FGF) as a prototype example for the interaction of heparin-binding cytokines with heparan sulfate proteoglycans is presented to illustrate the different levels of mutual dependence of the cytokine network and the ECM.


*Developmental Immunology*, 2000, Vol. 7(2-4), pp. 89-101
Reprints available directly from the publisher
Photocopying permitted by license only

© 2000 OPA (Overseas Publishers Association)
N.V. Published by license under the
Harwood Academic Publishers imprint,
part of the Gordon and Breach Publishing Group.
Printed in Malaysia

### Extracellular Matrix and Cytokines: A Functional Unit

ELKE SCHÖNHERR* and HEINZ-JÜRGEN HAUSSER

*Institute of Physiological Chemistry and Pathobiochemistry University of Münster, Waldeyerstrasse 15, D-48149 Münster, Germany*

The extracellular matrix (ECM) as well as soluble mediators like cytokines can influence the behavior of cells in very distinct as well as cooperative ways. One group of ECM molecules which shows an especially broad cooperativety with cytokines and growth factors are the proteoglycans. Proteoglycans can interact with their core proteins as well as their glycosaminoglycan chains with cytokines. These interactions can modify the binding of cytokines to their cell surface receptors or they can lead to the storage of the soluble factors in the matrix. Proteoglycans themselves may even have cytokine activity. In this review we describe different proteoglycans and their interactions and relationships with cytokines and we discuss in more detail the extracellular regulation of the activity of transforming growth factor-β (TGF-β) by proteoglycans and other ECM molecules. In the third part the interaction of heparan sulfate chains with fibroblast growth factor-2 (FGF-2, basic FGF) as a prototype example for the interaction of heparin-binding cytokines with heparan sulfate proteoglycans is presented to illustrate the different levels of mutual dependence of the cytokine network and the ECM.

*Keywords:* proteoglycan, glycosaminoglycan, TGF-β, FGF-2

*Abbreviations:*CSF-1, macrophage-colony stimulating factor, ECM, extracellular matrix, EGF, epidermal growth factor, FGF-2, fibroblast growth factor-2, GAG, glycosaminoglycan, GPI, glycosyl phosphatidy inositol, LAP, latency associated protein, LTBP, latent TGF-β binding protein, PDGF, platelet-derived growth factor, PG-100, proteoglycan-100, TGF-β, transforming growth factor-β

### INTRODUCTION

During the last few years it has become more and more apparent that the biological activities of cytokines can not be sufficiently described by their interactions with the corresponding signaling receptors alone. Instead, it appears that in many, if not most cases cytokines and the extracellular matrix (ECM) cooperate in forming an "information network" that regulates such fundamental processes as cell proliferation, differentiation and apoptosis.

The ECM is a complex supramolecular structure composed of different types of macromolecules which are predominantly linked by non-covalent bonds. The major constituents are the collagens, the non-collagenous glycoproteins, elastin, hyaluronan and the proteoglycans. With the exception of elastin (and hyaluronan), all the other classes consist of dif-

* Correspondence: Dr. Elke Schönherr, Institute of Physiological Chemistry and Pathobiochemistry, Waldeyerstrasse 15, D-48149 Münster, Germany. Phone: +49–251–8355586, Fax: +49–251–8355596, E-mail: schonhe@uni-muenster.de ferent families of related proteins which are derived from individual genes. They can be expressed in a tissue specific and developmentally distinct manner and can therefore form matrices with particular physical as well as biological properties which are tailored for their distinct biological functions as well as for specific interactions with the embedded cells. These interactions are mediated by cell surface receptors for matrix proteins which can be integrins (Hynes, 1992) or non-integrin-receptors (Shrivastava et al., 1997). The activation of these receptors by the ECM can influence the intracellular signal transduction and the expression of genes. By these means the matrix directly participates in the control of cell proliferation and differentiation as well as the survival of the cells (Frisch and Ruoslahti, 1997).

Another important factor in the control of cell behaviour are soluble mediators like cytokines. For the purpose of this review we want to use this term not only for the classical cytokines but also for growth factors, because similar rules apply for their interactions with ECM molecules. The relationships between cytokines and the extracellular matrix are manyfold. (1) Cytokines can influence the expression (Kovacs and DiPietro, 1994; Grande et al., 1997) and the turnover (Galis et al., 1994; 1995) of specific ECM molecules. (2) Certain matrix derived peptides can mediate the synthesis of cytokines (Lopez-Moratalla et al., 1995). (3) Cytokines can be dependent on ECM molecules as co-receptors (Rapraeger et al., 1991; Yayon et al., 1991) or (4) matrix cell surface receptors like integrins may be needed for the clustering of cytokine receptors to cause an effective signal transduction (Schneller et al., 1997). (5) Cytokines can use intracellular signal transduction pathways that are similar to the ways activated by matrix receptors (Schlaepfer et al., 1994; Short et al., 1998). (6) Some cytokines can directly bind to specific ECM constituents whereby their effects are localized to specific areas and/or they may be stored in the matrix for later release. In this review proteoglycans as the principal mediators of ECM cytokine interactions will be introduced and the interplay between these two systems will be characterized more closely in two well studied examples, TGF-β and FGF-2.

### PROTEOGLYCANS

Most of the interactions of cytokines with ECM molecules are mediated by proteoglycans. Proteoglycans are a heterogenous group of macromolecules characterized by at least one glycosaminoglycan (GAG) chain attached to a core proteins. GAG chains are unbranched, acidic heteropolysaccharides consisting, in principle, of repeating disaccharide units. On the basis of the constituting disaccharide units, three different types of sulfated GAGs can be distinguished: (1) chondroitin/dermatan sulfate, (2) heparan sulfate/heparin and (3) keratan sulfate. The backbone of chondroitin sulfate chains is built by disaccharide units of N-acetyl galactosamine and glucuronic acid residues that can be sulfated in the C4- and/or C6-position of the N-acetyl galactosamine residues. In dermatan sulfate the glucuronic acid is additionally epimerized to iduronic acid. The initial polysaccharide backbone of heparan sulfate and heparin consists of alternating N-acetyl glucosamine and glucuronic acid residues. This structure subsequently becomes modified by a series of reactions, each one creating the substrate structure for the next modifying step. The first modification is N-deacetylation and subsequent N-sulfation of N-acetyl glucosamine residues, both reactions being carried out by the same enzyme. In heparan sulfate these reactions are restricted to characteristic domains, leaving parts of the chain essentially unmodified, whereas in heparin these modifications run more towards completion. Subsequent modifications include the epimerization of glucuronic acid to iduronic acid, the sulfation of 2O of glucuronic acid (rare) and iduronic acid residues and the sulfation of 6O and 3O (rare) of glucosamine residues. The consequence of the incompleteness of each modifying step is the generation of an enormous structural heterogeneity in the modified domains of the heparan sulfate chain, leading to the potential for a great variety of specific interactions (Hardingham and Fosang, 1992). Finally, keratan sulfate chains are composed of alternating N-acetyl glucosamine and galactose residues that can be O-sulfated at C6 of either sugar (Fig. 1).

TABLE I Classification of Proteoglycans

| *Cell associated proteoglycans* | *Matrix associated proteoglycans* |
|---|---|
| *Proteoglycans with transmembrane domains*: | *Large proteoglycans*: |
| **Syndecans**[a]: | **Proteoglycans of the basal lamina**: |
| Syndecan-1 (Syndecan) | Perlecan[a] |
| Syndecan-2 (Fibroglycan) | Agrin[a] |
| Syndecan-3 (N-Syndecan) | Bamacan |
| Syndecan-4 (Ryudocan, Amphiglycan) | |
| | **Proteoglycans of the aggrecan-family**[a]: |
| **others**: | Aggrecan |
| Betaglycan[a] (TGF-β-Receptor III) | Versican |
| NG2[a] | Neurocan |
| CD44 | Brevican |
| | **others**: |
| *Proteoglycans with glycolipid-anchors*: | Collagens, Type IX, XII, XIV, XVIII |
| | Testican |
| **Glypicans**[a]: | Phosphacan |
| Glypican-1 (Glypican) | |
| Glypican-2 (Cerebroglycan) | |
| Glypican-3 (OCI-5) | *Small proteoglycans*: |
| Glypican-4 (K-Glypican) | |
| Glypican-5 | **Leucine-rich repeat proteoglycans**: |
| Glypican-6 | Decorin[a] |
| | Biglycan[a] |
| | Fibromodulin[a] |
| | Lumican[a] |
| | PRELP |
| | Keratocan |
| | Epiphycan (PG-Lb) |
| | Mimecan (Osteoglycin) |
| | **others**: |
| | PG-100/CSF-1 (M-CSF)[a] |

a. Proteoglycans, mentioned in the text.

There are many different types of proteoglycan core proteins in vertebrates. A complete discussion of all the different molecules and their proposed functions would be beyond the scope of this review. Therefore, only those proteoglycans which have been implicated in interactions with cytokines shall be described in more detail (Table I). In general, proteoglycans can be classified in cell associated molecules and matrix associated molecules. Some cell associated proteoglycans are inserted into the plasma membrane by a transmembrane domain, as are the members of the syndecan family (Carey, 1997), betaglycan (Lopez-Casillas et al., 1991), the proteoglycan NG2 (Nishiyama et al., 1991) and CD44 (Lesley et al., 1997). In contrast, members of the glypican family are anchored in the membrane via a glycosyl phosphatidyl inositol (GPI) moiety (Lander et al., 1996).

Almost all cells possess at their surface membrane-associated heparan sulfate proteoglycans that belong either to the syndecan or to the glypican family. In addition, betaglycan and certain splice variants of CD44 can carry heparan sulfate chains. Via their heparan sulfate chains, these molecules are able to interact with a variety of extracellular ligands, such as cytokines and growth factors, molecules of the surrounding extracellular matrix, or surface proteins of neighboring cells. Simultaneous binding of growth factors/cytokines to heparan sulfate chains and to the respective signaling receptor is the basis of the dual receptor theory for cytokine signaling that has originally been described for fibroblast growth factor-2 (FGF-2 or bFGF, Yayon et al., 1991; Rapraeger et al., 1991). However, the underlying principle appears to be valid for numerous other growth factors and cytokines as well (Selleck, 1998).

In addition to GAG mediated interactions, some of the membrane-associated proteoglycans have been shown to bind ligands specifically via their core proteins. Even though transforming growth factor-β (TGF-β) can interact with heparan sulfate chains, binding of TGF-β to betaglycan is mediated by the core protein of this proteoglycan (Andres et al., 1992). TGF-β bound to the betaglycan core protein can be presented to the TGF-β type II receptor which is involved in signal transduction (Lopez-Casillas et al., 1993). Another example is provided by the chondroitin sulfate proteoglycan NG2, which modulates the biological activity of platelet-derived growth factor AA (PDGF-AA) by interacting with the PDGF-α receptor (Grako and Stallcup, 1995). Indeed, NG2 has to be present for effective signal transduction via the PDGF-α receptor, as this receptor cannot be autophosphorylated in cells from NG2 (-/-) mice (Grako et al., 1999).

The matrix associated proteoglycans can be classified according to size, distribution and sequence similarities of their core proteins (Table I). Perlecan and agrin are large modular proteoglycans which are integrating constituents of basement membranes. The modular structure of their core proteins allows them to interact with a variety of other components of the basement membrane, thus contributing to the structural integrity of this specialized type of ECM. With the negative charge of their heparan sulfate chains, they contribute to the charge selectivity of the glomerular basement membrane. Additionally, these chains can interact with heparin-binding cytokines, mediating storage of these cytokines in the ECM (Iozzo, 1994).

Another group of large matrix associated proteoglycans, that are not constituents of basement membranes, are the members of the aggrecan family: aggrecan, versican, neurocan and brevican. They possess a binding site for hyaluronan at the N-terminus of their core protein and a lectin-like domain at the C-terminus. Therefore, they are suited to form a link between hyaluronan in the ECM and glycoproteins and glycolipids on the cell surface (Miura et al., 1999). In addition, they contain EGF-like domains which have been shown to mediate cell proliferation in fibroblasts (Zhang et al., 1998). Apparently, these matrix molecules exhibit cytokine-like activities by themselves.

The largest group of the matrix associated proteoglycans are the small leucine-rich repeat proteoglycans. Eight different members of this family have been described so far. As in other proteins containing the leucine-rich repeat motif, this structure is expected to mediate protein-protein interactions in these proteoglycans, too (Kresse et al., 1993; Iozzo, 1997; Hocking et al., 1998). The prototype member of the leucine-rich repeat proteoglycans is decorin which has a core protein of about 36 kDa, either two or three N-linked oligosaccharides and a single chondroitin/dermatan sulfate chain in mammals. Decorin received its name because it binds to the surface of collagen fibrils, thus "decorating" the fibrils. In addition to binding to different types of collagen, it can also interact with a variety of other ECM molecules, such as fibronectin and thombospondin, as well as with C1q and with different members of the TGF-β family. Whereas most of these interactions are mediated by the core protein, the GAG chain is also able to interact with other molecules, such as with heparin cofactor II (Whinna et al., 1993). In spite of these

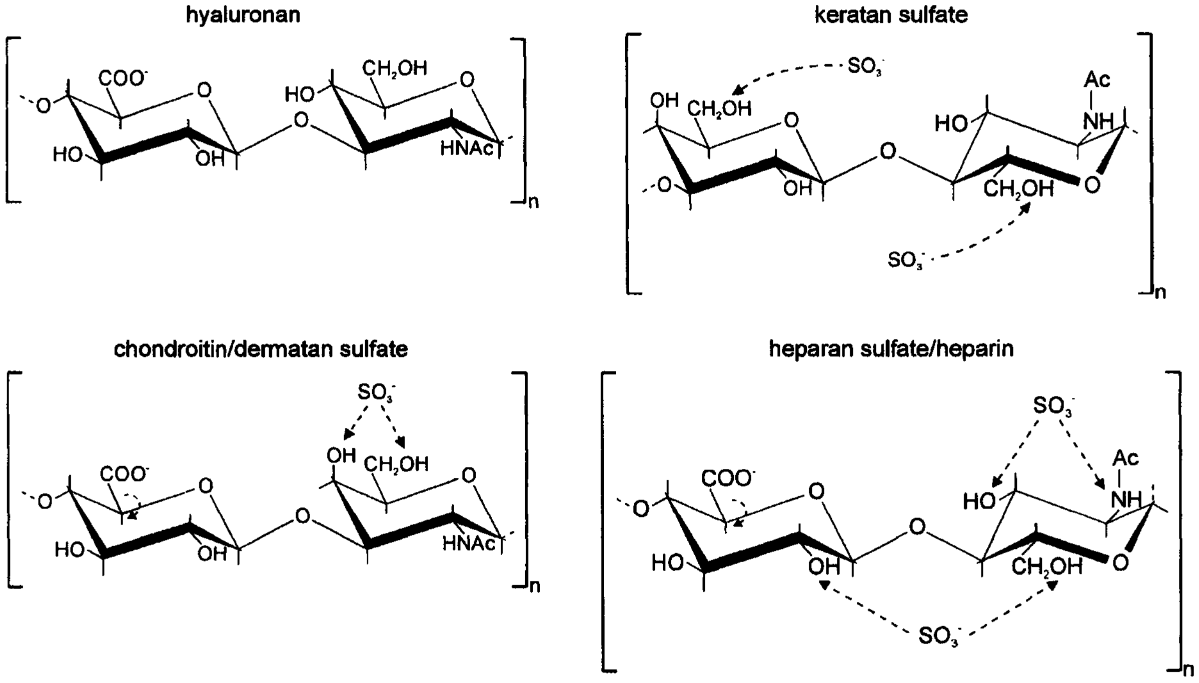

FIGURE 1 Disaccharide units of different glycosaminoglycans and their modifications many different interaotions, the decorin (-/-) mouse exhibits only a mild phenotype with reduced tensile strength of collagen fibrils from skin, suggesting that decorin is only essential for the formation of functional collagen fibrils in the skin, whereas in other organs the lack of this proteoglycan can be compensated, presumably by other members of the small leucine-rich proteoglycan family (Danielson et al., 1997). More recently it has been shown that decorin can bind to the EGF receptor which, after phosphorylation, causes an up-regulation of $p21^{WAF-1/CIP-1}$ and growth arrest in tumor cells, suggesting a further role for decorin in growth control (Moscatello et al., 1998). Apparently, this observation is not restricted to tumor cells, as endothelial cells induced to express decorin by infection with a replication deficient adenovirus containing the human decorin cDNA show a similar up-regulation of this inhibitor of cyclin dependent kinases (E. Schönherr, unpublished result). The closest relative of decorin is biglycan, which, however, can carry either one or two chondroitin/dermatan sulfate chains due to the presence of a second GAG attachment site. Biglycan, too, can interact with several types of collagen as well as with TGF-β. As decorin can compete with the binding of biglycan to collagen type I fibrils (Schönherr et al. 1995) and to TGF-β (Hildebrand et al., 1994), apparently both proteoglycans interact with the same or very close binding site on these molecules. In addition, both proteoglycans compete for the same binding site on the decorin/biglycan endocytosis receptor (Hausser et al., 1998). Core protein-mediated binding to this receptor, which additionally interacts with heparan sulfate chains (Hausser and Kresse, 1991; Hausser et al., 1993), is the prerequisite for internalization and subsequent intralysosomal degradation of decorin and biglycan by mesenchymal cells. Even though there are many similarities between these two small proteoglycans, the phenotype of the biglycan (-/-) mouse is quite different from that of the decorin (-/-) mouse. In contrast to skin fragility as a consequence of lack of decorin, lack of biglycan leads to osteoporosis (Xu et al., 1998). Fibromodulin and lumican are two other small leucine-rich repeat proteoglycans, which in contrast to decorin and biglycan can carry keratan sulfate chains. They too bind to collagen type I fibrils, albeit at different binding sites. Both, the fibromodulin (-/-) mouse (Swensson et al,. 1999) and the lumican (-/-) mouse (Chakravarti et al., 1998) have thinner and disorganized collagen fibrils, indicating that decorin, fibromodulin and lumican all are important for collagen fibrillogenesis.

Another small matrix proteoglycan, which however does not belong to the family of small leucine-rich proteoglycans, is proteoglycan-100 (PG-100). This chondroitin sulfate proteoglycan has been named according to the apparent molecular mass of its core protein (Schwarz et al., 1990). Later it was identified as the proteoglycan form of macrophage-colony stimulating factor (CSF-1, Price et al., 1992, Suzu et al., 1992). PG-100 is synthesized by many different cells like monocytes/macrophages (Chang et al., 1998), endothelial cells (Nelimarkka et al., 1997) and osteoblasts (Felix et al., 1996). Many other cells have been shown to synthesize CSF-1, but it has not been determined whether the cytokine is released in its proteoglycan form or in its mature form, which is a homodimer of 85 kDa. This homodimer is generated by partial proteolysis of the C-terminus which contains the GAG chain. It has not been clarified so far whether this cleavage reaction occurs due to an autocatalytic activity of PG-100 itself, or whether other proteases are involved in this step. Comparison of the growth stimulatory effect of PG-100 and the mature CSF-1 showed that the proteoglycan form is less active (Partenheimer et al., 1995). Therefore, the proteoglycan form could be a storage form of the cytokine which is bound via its GAG chain to ECM molecules (Suzu et al., 1992, Ohtsuki et al., 1993) and which can be released by proteolytic degradation of the matrix. In addition, the proteoglycan form but not the mature form can bind FGF-2. Peptides of the core protein of PG-100, which mediate this binding, can inhibit the growth-stimulatory effect of FGF-2 (Suzu, et al., 1997). It therefore appears that PG-100 is not only a constituent of the ECM, but also a regulator of cytokine action and, in its mature form, a cytokine by itself.

### THE EXTRACELLULAR REGULATION OF TGF-β ACTIVITY

The TGF-β superfamily consists of a large number of different polypeptide factors, comprising not only the different TGF-βs themselves, but also several bone morphogenetic proteins and growth differentiation factors. These molecules are involved in the regulation of such diverse processes as cell proliferation, differentiation, adhesion, migration and survival. Three members of the TGF-β subfamily are found in mammalian cells. However, most of the studies have been done with TGF-β 1 and 2 (Massagué, 1998). The importance of TGF-β 1 for the immune system has become especially evident when TGF-β 1 (-/-) mice were generated by targeted gene disruption. Only about half of these mice were normally born and two weeks after birth they developed a wasting syndrome with multiple inflammatory lesions in almost all organs without exposure to a pathogen, indicating an autoimmune reaction (Kulkarni et al., 1995; Boivin et al., 1995). In contrast, all the TGF-β 2 (-/-) mice died perinatally and had multiple developmental defects which did not overlap with the TGF-β 1 (-/-) mice, indicating different functions of these two isoforms during development (Sanford et al., 1997). This observation is even more interesting as the two isoforms, when applied to cultured cells, very often lead to similar reactions, indicating that under cell culture conditions important modulating factors are missing. These factors may be *in vivo* supplied by components of the surrounding extracellular environment of the cells.

The active form of the TGF-βs is a disulfide-linked homodimer. This molecule is synthesized in a pre-pro-form. The pro-peptide is cleaved off before secretion, but remains associated with the homodimer as latency associated protein (LAP), thereby keeping the molecule in a biologically inactive state. In addition, LAP mediates the binding to latent TGFβ binding proteins (LTBPs) by disulfide bonds. Four different LTBPs as well as two splice variants have been identified so far. These multidomain glycoproteins belong to the fibrillin superfamily. They contain epidermal growth factor (EGF)-like domains, most of which are able to bind $Ca^{2+}$, many cysteine-rich domains and hybrid motifs which contain features of both domains (Sinha et al., 1998). LTBPs with or without bound latent TGF-β complex can be covalently crosslinked by transglutaminase reactions to form fibrillar structures. In addition, they can be bound to other proteins of the ECM like collagens and fibronectin. In microfibrils, LTBPs have been found associated with other members of the fibrillin superfamily. The exact mode of association with these structures, however, is not known. In addition to LTBPs, other proteins can bind LAP, as e.g. a 140 kDa-protein which is homologous to chicken cysteine-rich fibroblast growth factor receptor. This molecule was found in CHO cells transfected with TGF-β 1, where the latent complex was in part associated with LTBP-1 and in part with this protein (Olofsson et al., 1997). The small leucine-rich repeat proteoglycan fibromodulin, too, has been shown to bind the TGF-β LAP complex (Hildebrand et al., 1994). Apparently, this complex can associate with different structures within the ECM, leading to the temporary deposition of latent TGF-β waiting for activation.

Activation of TGF-β in such complexes is very likely achieved by limited proteolysis mediated by serine proteases, like plasmin, thrombin, elastase or chymase which degrade LAP and the associated proteins to release activated TGF-β, e.g. during ECM remodeling (Munger et al., 1997). Another mode of activation involves thrombospondin, which specifically binds LAP, thus inhibiting the reformation of the latent complex (Ribeiro et al., 1999). The integrin $\alpha_v\beta_6$ also can bind LAP and it has been shown that cells expressing this integrin can activate TGF-β (Munger et al., 1999). In vitro, TGF-β can be activated not only by proteases, but also by acid, alkali, heat or glycosidases.

Signal transduction of TGF-β requires sequential binding of activated TGF-β to the type II and the type I receptor to form a heterotetrameric complex which has intracellular serine/threonine kinase activity. Two other receptors for TGF-β have been found on the cell surface, betaglycan and endoglin (CD105). Apparently, these receptors do not have a signaling function. Betaglycan is a so called part time proteoglycan which can carry chondroitin and/or heparan sulfate chains or no GAG chains at all. The core protein has two separate binding sites for TGF-β, one at the N-terminus and the other at the C-terminus of the extracellular domain (Kaname and Ruoslahti, 1996). Bound TGF-β, especially TGF-β2 can be presented to the type II receptor (Lopez-Casillas et al., 1993). In this way betaglycan can assist the two signaling receptors in the formation of the active signal transducing complex. By shedding the extracellular domain of this proteoglycan from the cell surface, free soluble betaglycan may, on the other hand, function as a receptor antagonist which keeps TGF-β away from the signaling receptors (Lopez-Casillas et al., 1994). The other accessory receptor, endoglin, shares some sequence similarity with betaglycan. It occurs on the cell surface as a disulfide linked dimer. In contrast to betaglycan, this molecule only binds TGF-β1 and 3 but not TGF-β2 (Cheifetz et al., 1992). In more recent studies differences between the two assisting receptors in the presentation of TGF-β to the signaling receptors have been shown, too. In a myoblast system endoglin facilitates the binding of TGF-β to both the type I and type II receptors, whereas betaglycan only assisted in binding to the type II receptor (Letamendia et al., 1998). Whether this finding is of general importance or specific for myoblasts, remains to be determined.

Not only cell surface proteins and proteoglycans but also molecules of the surrounding ECM can bind active TGF-β. Decorin, biglycan and fibromodulin all have been shown to bind this cytokine (Hildebrand et al., 1994). All these three small proteoglycans appear to compete with betaglycan for the same binding site on the TGF-β dimer (Fukushima et al., 1993). However, the consequences of this interaction is still a matter of debate. Whereas some investigators suggested a direct inactivation of TGF-β by decorin (Yamaguchi and Ruoslahti, 1988; Yamaguchi et al., 1990), others found no change in TGF-β activity (Hausser et al., 1994) or even an activation of TGF-β (Takeuchi et al., 1994) due to complex formation with decorin. One possible explanation for these conflicting results in different experimental settings may be further interactions with yet unidentified binding partners which may modify the interaction of decorin,

TGF-β and the TGF-β receptors. It could be shown, for instance, that decorin bound to collagen type I fibrils is still able to bind TGF-β (Hausser et al., 1994, Schönherr et al., 1998). This suggests that in vivo decorin could inhibit TGF-β by immobilizing the cytokine in the ECM, keeping it away from its signaling receptors on the cell surface. This mechanism for regulating TGF-β activity is further corroborated by experiments showing that osteosarcoma cells (MG-63) transfected with a decorin sense construct and cultured in a collagen type I lattice exhibit a reduced reactivity to TGF-β than the same cells transfected with a decorin anti-sense construct, which causes a lower expression of endogenous decorin (A. Markmann, personal communication). Immobilized TGF-β might subsequently become "activated" by partial proteolysis of decorin, as it has been shown that decorin is a substrate for some metalloproteinases (Imai et al., 1997), or by degradation of the collagen matrix. Another possible binding partner is dermatopontin, an ECM constituent that has been shown to bind decorin, collagen type I fibrils and TGF-β. In a recent study it could be demonstrated that dermatopontin can enhance the binding of TGF-β to its cell surface receptors, thus leading to an increased signal transduction (Okamoto et al., 1999). Finally, an interaction of decorin with its endocytosis receptor (Hausser et al., 1989) present at the cell surface of many cells could be envisaged as a further possibility for decorin to influence TGF-β activity. If, for instance, the decorin/TGF-β complex would be a substrate for endocytosis, decorin could mediate the clearance of extracellular TGF-β, thereby decreasing its activity. Whether the decorin/TGF-β complex is able to bind to the endocytosis receptor has so far not been investigated. However, the observation that both, binding of decorin to its receptor and binding of TGF-β to decorin can be inhibited by an antibody directed against the central region of the decorin core protein (aa 155–260 of pre-pro decorin) argues for a close spatial relationship of the two binding sites (Hausser et al., 1998; Schönherr et al., 1998).

Not only the activity of TGF-β is affected by molecules of the ECM, TGF-β itself has a profound effect on the synthesis and degradation of a large number of ECM molecules. A stimulation of the synthesis of fibronectin, different types of collagen, thrombospondin, laminin, some proteoglycans, tissue inhibitors of metalloproteinases, integrins etc. as well as the inhibition of the expression of metalloproteinases together lead to an increased deposition of ECM (Massagué, 1990). Such an increased ECM deposition by resident and/or invading cells is necessary during wound healing (Sporn and Roberts, 1992). In chronic inflammation, however, excessive ECM synthesis can lead to fibrosis with concomitant destruction of normal organ function (Okuda et al., 1990; Border and Noble, 1994). Therefore, during the last decade strategies to interfere with the TGF-β mediated ECM deposition have attracted a lot of interest. In this context the TGF-β-binding small leucine-rich proteoglycans have been discussed as potential therapeutics in fibrotic diseases, as intravenous application of exogenous decorin (Border et al., 1992) as well as a gene therapeutic approach with decorin cDNA (Isaka et al., 1996) led to a reduction in TGF-β mediated matrix deposition and to an amelioration of the symptoms in an acute model of mesangioproliferative glomerulonephritis. In addition to mere binding of TGF-β, it has been found recently that decorin can also abrogate the expression of TGF-β1 and 2 (Stander et al., 1998). Furthermore, these authors demonstrated that decorin can lead to an increased invasion of B- and T-cells, in contrast to TGF-β which is a strong chemoatractant for monocytes (Wahl et al., 1987). Such a change in the type of the invading cells could change the whole character of the inflammation. Finally, by binding to fibronectin, decorin could exert an antiadhesive effect on invading cells (Winnemöller et al., 1991), thus decreasing the population of cells that contribute to the matrix deposition under the influence of TGF-β. Obviously, several different mechanisms could be involved in mediating the antifibrotic activity of decorin.

### FIBROBLAST GROWTH FACTOR-2 AND THE ECM

Whereas most of the interactions of TGF-β with the ECM are mediated by proteins and the significance of its interaction with heparan sulfate chains remain to be elucidated, binding to heparan sulfate chains is of outstanding importance for FGF-2. This cytokine belongs to an ever increasing family of growth factors with until now 18 known members (Fernig and Gallagher, 1994; Ohbayashi et al., 1998). It plays part in such important physiological processes as embryonic development, angiogenesis, neuronal differentiation and wound repair, being involved in the regulation of migration, proliferation and differentiation of numerous cell types. Even though it shares only 10% sequence identity with interleukin 1β, the tertiary structure of these two molecules is identical (Zhang et al., 1991). The relationship between the "growth factor" FGF-2 and "classical" cytokines, however, is not only a structural one. In concert with other soluble factors, it participates in positively regulating hematopoesis by acting on stromal cells as well as on early and committed hematopoetic progenitors, preventing apoptosis and leading to an increased cell proliferation and cytokine secretion (Allouche, 1995). This is achieved by acting synergistically with numerous hematopoetic cytokines as well as by antagonizing the effects of TGF-β.

Members of the FGF family exert their biological effects by binding to four structurally related high-affinity FGF receptors (Friesel and Maciag, 1995; Klint and Claesson-Welsh, 1999). These polypeptides contain an extracellular domain composed of up to three immunoglubulin-like domains, a transmembrane domain and an intracellular tyrosine kinase domain. By alternative splicing, structural variants are generated that differ in their ligand-binding specificities and affinities. In addition to these high-affinity signaling receptors, it has been recognized for long time that FGF-2 (and the other members of the FGF family) binds with lower affinity to heparan sulfate chains present in the ECM and on the cell surface. This interaction not only leads to a sequestration of the cytokine within the ECM and to its stabilization and protection from inactivation (Vlodavsky et al., 1996). It is also required for the cytokine to exert its biological activity (Yayon et al., 1991; Rapraeger et al., 1991).

The minimal binding structure on the heparan sulfate chain has been revealed to be a pentasaccharide containing at least one iduronic acid residue sulfated at C2 and one or two N-sulfate groups (Maccarana et al., 1993; Faham et al., 1996). Additional sulfate groups, either at C2 of iduronic acid or at C6 of glucosamine, are not required for binding and do not interfere with binding. For stimulation of the mitogenic activity of FGF-2, however, a sequence of at least 12 saccharides containing sulfate groups at C2 of iduronic acid as well as at C6 of glucosamine is necessary (Guimond et al., 1993).

Different models have been discussed to explain the activation of FGF-2 by interaction with heparan sulfate: (1) binding of heparan sulfate leads to an altered conformation of the cytokine, enabling it to interact efficiently with its signaling receptor; (2) binding of two FGF-2 molecules on the same heparan sulfate chain facilitates receptor dimerization necessary for signal transduction by presenting a "dimeric" ligand and (3) formation of a ternary complex by simultaneous binding of ligand and receptor to adjacent binding sites on the same heparan sulfate chain (Turnbull and Gallagher, 1993; Salmivirta et al., 1996). Indeed, the receptor has been shown to be able to interact with heparan sulfate (Kan et al., 1993), and this interaction can lead to an activation of the receptor, even in the absence of cytokine (Gao and Goldfarb, 1995). The observation of additional structural requirements for activation in comparison with FGF-2 binding alone is in support of the third model, postulating the formation of a ternary complex as the signaling complex. There are two important predictions derived from this model: (1) depending on the spacing of the two binding sites for receptor and FGF-2 on the GAG chain, interaction of the cytokine with heparan sulfate will either promote or inhibit its cellular response and (2) the fine structure of the heparan sulfate chain will determine which of the heparin-binding cytokines will be activated, provided that different cytokines possess different binding requirements. Indeed, different fine structures appear to be necessary for activation of FGF-1, FGF-2 and FGF-4 (Guimond et al., 1993). Therefore, by changing the fine structures of their cell surface heparan sulfates, cells may select the cytokine to be activated. This might be achieved either by substituting existing heparan sulfate proteoglycans with newly synthesized ones or by modification of the GAG chains during recycling of proteoglycans (Fransson et al., 1995). In the developing neuroepithelium, cell surface associated heparan sulfate proteoglycans carry chains that are able to bind and activate FGF-2 on day 9 (Nurcombe et al., 1993). Two days later, with the onset of FGF-1 expression, the fine structure of newly synthesized heparan sulfate chains is changed, allowing binding and activation of FGF-1. At this time, the more differentiated cells synthesize longer heparan sulfate chains containing more modified domains with a higher content of disaccharide units sulfated at C2 of the iduronic acid and, importantly, at C6 of the glucosamine residues (Brickman et al., 1998a; Brickman et al., 1998b). Sulfation at C6 of glucosamine appears to be required for binding of FGF-1 (Fromm et al., 1997).

Obviously, due to the fact that cytokine activation requires the formation of a ternary complex of cytokine, receptor and heparan sulfate, "activating" heparan sulfate chains have to be in close proximity of the receptor molecules. Therefore, chains of matrix associated proteoglycans that are remote from the signaling receptors will sequester the cytokine within the ECM, actually leading to its inactivation, provided they possess the appropriate binding structures. Upon proteolytic degradation of the matrix or upon degradation of the heparan sulfate chains by released heparanases, these bound cytokines however may become available for signaling. On the contrary, cytokine bound to cell surface associated heparan sulfate proteoglycans may become inactivated due to the shedding of the respective proteoglycan molecules, either by proteolytic cleavage of a juxtamembraneous cleavage site in the case of syndecans or by cleavage of the GPI anchor in the case of the glypicans. Thus, in addition to factors controlling release and degradation, a complex interplay involving the regulated synthesis and distribution of heparan sulfate proteoglycans as well as the regulated activities of extracellular enzymes acting on these proteoglycans determines the availability and biological activity of heparan-sulfate binding cytokines.

### *References*

Allouche, M. and Bikfalvi, A. (1995) The role of fibroblast growth factor-2 (FGF-2) in hematopoiesis. Prog. Growth Factor Res. 6:35–48.

Andres, J.L., DeFalcis, D., Noda, M. and Massagué, J. (1992) Binding of two growth factor families to separate domains of the proteoglycan betaglycan. J. Biol. Chem. 267:5927–5930.

Boivin, G.P., O'Toole, B.A., Orsmby, I.E., Diebold, R.J., Eis, M.J., Doetschman and T., Kier, A.B. (1995) Onset and progression of pathological lesions in transforming growth factor-β 1-deficient mice. Am. J. Pathol. 146:276–288.

Border, W.A. and Noble, N.A. (1994) Transforming growth factor-β in glomerular injury. Exp. Nephrol. 2:13–17.

Border, W.A., Noble, N.A., Yamamoto, T., Harper, J.R., Yamaguchi, Y., Pierschbacher, M.D. and Ruoslahti, E. (1992) Natural inhibitor of transforming growth factor-β protects against scarring in experimental kidney disease. Nature 360:361–364.

Brickman, Y.G., Ford, M.D., Gallagher, J.T., Nurcombe, V., Bartlett, P.F. and Turnbull, J.E. (1998a) Structural modification of fibroblast growth factor-binding heparan sulfate at a determinative stage of neural development. J. Biol. Chem. 273:4350–4359.

Brickman, Y.G., Nurcombe, V., Ford, M.D., Gallagher, J.T., Bartlett, P.F. and Turnbull, J.E. (1998b) Structural comparison of fibroblast growth factor-specific heparan sulfates derived from agrowing or differentiating neuroepithelial cell line. Glycobiology 8:463–471.

Carey, D.J. (1997) Syndecans: multifunctional cell surface receptors. Biochem. J. 327:1–16.

Chakravarti, S., Magnuson, T., Lass, J.H., Jepsen, K.J., LaMantia, C. and Carroll, H. (1998) Lumican regulates collagen fibril assembly: skin fragility and corneal opacity in the absence of lumican. J. Cell Biol. 141:1277–1286.

Chang, M.Y., Olin, K.L., Tsoi, C., Wight, T.N. and Chait, A. (1998) Human monocyte-derived macrophages secrete two forms of proteoglycan-macrophage colony-stimulating factor that differ in their ability to bind low density lipoproteins. J. Biol. Chem. 273:15985–15992.

Cheifetz, S., Bellon, T., Cales, C., Vera, S., Bernabeu, C., Massagué, J. and Letarte, M.J. (1992) Endoglin is a component of the transforming growth factor-β receptor system in human endothelial cells. Biol. Chem. 267:19027–30.

Danielson, K.G., Baribault, H., Holmes, D.F., Graham, H., Kadler, K.E. and Iozzo R.V. (1997) Targeted disruption of decorin leads to abnormal collagen fibril morphology and skin fragility. J. Cell Biol. 136:729–743.

Dickson, M.C., Martin, J.S., Cousins, F.M., Kulkarni, A.B., Karlsson, S. and Akhurst, R.J. (1995) Defective haematopoiesis and vasculogenesis in transforming growth factor-β 1 knock out mice. Development 121:1845–1854.

Faham, S., Hileman, R.E., Fromm, J.R., Linhardt, R.J. and Rees, D.C. (1996) Heparin structure and interactions with basic fibroblast growth factor. Science 271:1116–1120.

Felix, R., Halasy-Nagy, J., Wetterwald, A., Cecchini, M.G., Fleisch, H. and Hofstetter, W. (1996) Synthesis of membrane- and matrix-bound colony-stimulating factor-1 by cultured osteoblasts. J. Cell Physiol. 166:311–322.

Fernig, D.G. and Gallagher, J.T. (1994) Fibroblast growth factors and their receptors: an information network controlling tissue growth, morphogenesis and repair. Prog. Growth Factor Res. 5:353–377.

Fransson, L.A., Edgren, G., Havsmark, B. and Schmidtchen, A. (1995) Recycling of a glycosylphosphatidylinositol-anchored heparan sulphate proteoglycan (glypican) in skin fibroblasts. Glycobiology 5:407–415.

Friesel, R.E. and Maciag, T. (1995) Molecular mechanisms of angiogenesis: fibroblast growth factor signal transduction. FASEB J. 9:919–925.

Frisch, S.M. and Ruoslahti, E. (1997) Integrins and anoikis. Curr. Opin. Cell Biol. 9:701–6.

Fromm, J.R., Hileman, R.E., Weiler, J.M. and Linhardt, R.J. (1997) Interaction of fibroblast growth factor-1 and related peptides with heparan sulfate and its oligosaccharides. Arch. Biochem. Biophys. 346:252–262.

Fukushima, D., Butzow, R., Hildebrand, A. and Ruoslahti, E. (1993) Localization of transforming growth factor β binding site in betaglycan. Comparison with small extracellular matrix proteoglycans. J. Biol. Chem. 268:22710–22715.

Gao, G. and Goldfarb, M. (1995) Heparin can activate a receptor tyrosine kinase. EMBO J. 14:2183–2190.

Grako, K.A., Ochiya, T., Barritt, D., Nishiyama, A. and Stallcup, W.B. (1999) PDGF (α)-receptor is unresponsive to PDGF-AA in aortic smooth muscle cells from the NG2 knockout mouse. Cell Sci. 112:905–915.

Grande J.P., Melder D.C. and Zinsmeister A.R. (1997) Modulation of collagen gene expression by cytokines: stimulatory effect of transforming growth factor-β1, with divergent effects of epidermal growth factor and tumor necrosis factor-α on collagen type I and collagen type IV. J. Lab. Clin. Med. 130:476–86.

Guimond, S., Maccarana, M., Olwin, B.B., Lindahl, U. and Rapraeger, A.C. (1993) Activating and inhibitory heparin sequences for FGF-2 (basic FGF). Distinct requirements for FGF-1, FGF-2, and FGF-4. J. Biol. Chem. 268:23906–23914.

Hardingham, T.E. and Fosang, A.J. (1992) Proteoglycans: many forms, many functions. FASEB J. 6:861–870.

Harper, J.R., Spiro, R.C., Gaarde, W.A., Tamura, R.N., Pierschbacher, M.D., Noble, N.A., Stecker, K.K. and Border, W.A. (1994) Role of transfotming growth factor β and decorin in controlling fibrosis. Methods Enzymol. 245:241–254.

Hausser, H., Hoppe, W., Rauch, U. and Kresse, H. (1989) Endocytosis of a small dermatan sulphate proteoglycan. Identification of binding proteins. Biochem. J. 263:137–142.

Hausser, H. and Kresse, H. (1991) Binding of heparin and of the small proteoglycan decorin to the same endocytosis receptor proteins lead to different metabolic consequences. J. Cell Biol. 114:45–52.

Hausser, H., Witt, O. and Kresse, H. (1993) Influence of membrane-associated heparan sulfate on the internalization of the small proteoglycan decorin. Exp. Cell Res. 208:398–406.

Hausser, H., Gröning, A., Hasilik, A., Schönherr, E. and Kresse, H. (1994) Selective inactivity of TGF-β/decorin complexes. FEBS Lett. 353:243–245.

Hausser, H. Schönherr, E., Müller, M. Liszio, C., Zhao, B., Fisher, L.W. and Kresse, H. (1998) Receptor-mediated endocytosis of decorin: involvement of leucine-rich repeat structures. Arch. Biochem. Biophys. 349:363–370.

Hildebrand, A., Romaris, M., Rasmussen, L.M., Heinegard, D., Twardzik, D.R., Border, W.A. and Ruoslahti, E. (1994) Interaction of the small interstitial proteoglycans biglycan, decorin and fibromodulin with transforming growth factor β. Biochem. J. 302:527–534.

Hocking, A.M., Shinomura, T. and McQuillan, D.J. (1998) Leucin-rich repeat glycoproteins of the extracellular matrix. Matrix Biol. 17:1–19.

Hynes, R.O. (1992) Integrins: versatility, modulation and signaling in cell adhesion. Cell 69:11–25.

Imai, K., Hiramatsu, A., Fukushima, D., Pierschbacher, M.D. and Okada, Y. (1997) Degradation of decorin by matrix metalloproteinases: identification of the cleavage sites, kinetic analyses and transforming growth factor-β 1 release. Biochem. J. 322:809–814.

Iozzo, R..V. (1998) Matrix proteoglycans: from molecular design to cellular function. Annu. Rev. Biochem. 67:609–52.

Iozzo, R.V. (1994) Perlecan: a gem of a proteoglycan. Matrix Biology 14:203–208.

Iozzo, R.V. (1997) The family of small leucin-rich proteoglycans: key regulators of matrix assembly and cellular growth. Crit. Rev. Biochem. Mol. Biol. 32: 141–174.

Isaka, Y., Brees, D.K., Ikegaya, K., Kaneda, Y., Imai, E., Noble, N.A. and Border, W.A. (1996) Gene therapy by skeletal muscle expression of decorin prevents fibrotic disease in rat kidney. Nat. Med. 2:418–423.

Kann, M., Wang, F., Xu, J., Crabb, J.W., Hou, J. and McKeehan, W.L. (1993) An essential heparin-binding domain in the fibroblast growth factor receptor kinase. Science 259:1918–21.

Kaname, S. and Ruoslahti, E. (1996) Betaglycan has multiple binding sites for transforming growth factor-β 1. Biochem. J. 315:815–820.

Klint, P. and Claesson-Welsh, L. (1999) Signal transduction by fibroblast growth factor receptors. Front. Biosci. 4:D165–177.

Kovacs, E.J. and DiPietro L.A. (1994) Fibrogenic cytokines and connective tissue production FASEB J. 8:854–861.

Kresse, H., Hausser, H., and Schönherr, E. (1993) Small proteoglycans. Experientia 49:403–416.

Kulkarni, A.B., Ward, J.M., Yaswen, L., Mackall, C.L., Bauer, S.R., Huh, C.G., Gress, R.E. and Karlsson, S. (1995) Transforming growth factor-β1 null mice. An animal model for inflammatory disorders. Am. J. Pathol. 146:264–275.

Letamendia, A., Lastres, P., Botella, L.M., Raab, U., Langa, C., Velasco, B., Attisano, L. and Bernabeu, C. (1998) Role of endoglin in cellular responses to transforming growth factor-β. A comparative study with betaglycan. J. Biol. Chem. 273:33011–33019.

Lesley, J., Hyman, R., English, N., Catterall, J.B. and Turner, G.A. (1997) CD44 in inflammation and metastasis. Glycoconj. J. 14:611–622.

Lopez-Casillas, F., Cheifetz, S., Doody, J., Andres, J.L., Lane, W.S. and Massagué, J. (1991) Structure and expression of the membrane proteoglycan betaglycan, a component of the TGF-β receptor system. Cell 67:785–795.

Lopez-Casillas, F., Wrana, J.L. and Massagué, J. (1993) Betaglycan presents ligand to the TGF β signaling receptor. Cell 73:1435–1444.

Lopez-Casillas, F., Payne, H.M., Andres, J.L. and Massagué, J. (1994) Betaglycan can act as a dual modulator of TGF-β access to signaling receptors: mapping of ligand binding and GAG attachment sites. J. Cell Biol. 124:557–568.

Maccarana, M., Casu, B. and Lindahl, U. (1993) Minimal sequence in heparin/heparan sulfate required for binding of basic fibroblast growth factor. J. Biol. Chem. 268:23898–23905.

Massagué, J. (1990) The Transforming growth factor-β family. Annu. Rev. Cell Biol. 6:597–641.

Massagué, J. (1998) TGF-β signal transduction. Annu. Rev. Biochem. 67: 753–791.

Miura, R., Aspberg, A., Ethell, I.M., Hagihara, K., Schnaar, R.L., Ruoslahti, E. and Yamaguchi, Y. (1999) The proteoglycan lectin domain binds sulfated cell surface glycolipids and promotes cell adhesion. J. Biol. Chem. 274:11431–11438.

Moscatello, D.K., Santra, M., Mann, D.M., McQuillan, D.J., Wong, A.J. and Iozzo, R.V. (1998) Decorin suppresses tumor cell growth by activating the epidermal growth factor receptor. J. Clin. Invest. 101:406–412.

Munger, J.S., Harpel, J.G., Gleizes, P.E., Mazzieri, R., Nunes, I. and Rifkin, D.B. (1997) Latent transforming growth factor-β: structural features and mechanisms of activation. Kidney Int. 51:1376–1382.

Munger, J.S., Huang, X., Kawakatsu, H., Griffiths, M.J., Dalton, S.L., Wu, J., Pittet, J.F., Kaminski, N., Garat, C., Matthay, M.A., Rifkin, D.B. and Sheppard, D. (1999) The integrin $\alpha_v\beta_6$ binds and activates latent TGF β 1: a mechanism for regulating pulmonary inflammation and fibrosis. Cell 96:319–328.

Nelimarkka, L., Kainulainen, V., Schönherr, E., Moisander, S., Jortikka, M., Lammi, M., Elenius, K., Jalkanen, M. and Järveläinen, H.J. (1997) Expression of small extracellular chondroitin/dermatan sulfate proteoglycans is differentially regulated in human endothelial cells. Biol. Chem. 272:12730–12737.

Nishiyama, A., Dahlin, K.J., Prince, J.T., Johnstone, S.R. and Stallcup, W.B. (1991) The primary structure of NG2, a novel membrane-spanning proteoglycan. J. Cell Biol. 114:359–371.

Nurcombe, V., Ford, M.D., Wildschut, J.A. and Bartlett, P.F. (1993) Developmental regulation of neural response to FGF-1 and FGF-2 by heparan sulfate proteoglycan. Science 260:103–106.

Ohbayashi, N., Hoshikawa, M., Kimura, S., Yamasaki, M., Fukui, S. and Itoh, N. (1998) Structure and expression of the mRNA encoding a novel fibroblast growth factor, FGF-18. J. Biol. Chem. 273:18161–18164.

Ohtsuki, T., Suzu, S., Hatake, K., Nagata, N., Miura, Y. and Motoyoshi, K. (1993) A proteoglycan form of macrophage colony-stimulating factor that binds to bone-derived collagens and can be extracted from bone matrix. Biochem. Biophys. Res. Commun. 190:215–222.

Okamoto, O., Fujiwara, S., Abe, M. and Sato, Y. (1999) Dermatopontin interacts with transforming growth factor β and enhances its biological activity. Biochem. J. 337:537–541.

Okuda, S., Languino, L.R., Ruoslahti, E. and Border, W.A. (1990) Elevated expression of transforming growth factor-β and proteoglycan production in experimental glomerulonephritis. Possible role in expansion of the mesangial extracellular matrix. J. Clin. Invest. 86:453–462.

Olofsson, A., Hellman, U., Ten Dijke, P., Grimsby, S., Ichijo, H., Moren, A., Miyazono, K. and Heldin, C.H. (1997) Latent transforming growth factor-β complex in Chinese hamster ovary cells contains the multifunctional cysteine-rich fibroblast growth factor receptor, also termed E-selectin-ligand or MG-160. Biochem. J. 324:427–434.

Partenheimer, A., Schwarz, K., Wrocklage, C., Kölsch, E. and Kresse, H. (1995) Proteoglycan form of colony-stimulating factor-1 (proteoglycan-100). Stimulation of activity by glycosaminoglycan removal and proteolytic processing. J. Immunol. 155:5557–5565.

Price, L.K., Choi, H.U., Rosenberg, L. and Stanley, E.R. (1992) The predominant form of secreted colony stimulating factor-1 is a proteoglycan. J. Biol. Chem. 267:2190–2199.

Rapraeger, A.C., Krufka, A. and Olwin, B.B. (1991) Requirement of heparan sulfate for FGF-mediated fibroblast growth and myoblast differentiation. Science 252:1705–1708.

Ribeiro, S.M., Poczatek, M., Schultz-Cherry, S., Villain, M. and Murphy-Ullrich, J.E. (1999) The activation sequence of thrombospondin-1 interacts with the latency-associated peptide to regulate activation of latent transforming growth factor-β. J. Biol. Chem. 274:13586–13593.

Salmivirta, M., Lidholt, K. and Lindahl, U. (1996) Heparan sulfate: a piece of information. FASEB J. 10:1270–1279.

Sanford, L.P., Ormsby, I., Gittenberger-de Groot, A.C., Sariola, H., Friedman, R., Boivin, G.P., Cardell, E.L. and Doetschman, T. (1997) TGFβ2 knockout mice have multiple developmental defects that are non-overlapping with other TGFβ knockout phenotypes. Development 124:2659–2670.

Schlaepfer, D.D., Hanks, S.K., Hunter, T. and van der Geer P. (1994) Integrin-mediated signal transduction linked to Ras pathway by GRB2 binding to focal adhesion kinase. Nature 372:786–791.

Schneller M. Vuori K. and Ruoslahti E. (1997) $\alpha_v\beta_3$ integrin associates with activated insulin and PDGF β receptors and potentiates the biological activity of PDGF. EMBO J. 16:5600–5607.

Schönherr, E., Witsch-Prehm, P., Harrach, B., Robenek, H, Rauterberg, J., Kresse, H. (1995) Interaction of biglycan with type I collagen. J. Biol. Chem. 270:2776–2783.

Schönherr, E., Broszat, M., Brandan, E., Bruckner, P. and Kresse, H. (1998) Decorin core protein fragment Leu155-Val260 interacts with TGF-β but does not compete for decorin binding to type I collagen. Arch. Biochem. Biophys. 355:241–248.

Schwarz, K., Breuer, B. and Kresse, H. (1990) Biosynthesis and properties of a further member of the small chondroitin/dermatan sulfate proteoglycan family. J. Biol. Chem. 265:22023–22028.

Short S.M., Talbott G.A. and Juliano R.L. (1998) Integrin-mediated signaling events in human endothelial cells. Mol. Biol. Cell 9:1969–1980.

Shrivastava, A., Radziejewski, C., Campbell, E., Kovac, L., McGlynn, M., Ryan, T.E., Davis, S., Goldfarb, M.P., Glass, D.J., Lemke, G. and Yancopoulos, G.D. (1997) An orphan receptor tyrosine kinase family whose members serve as nonintegrin collagen receptors. Mol. Cell 1:25–34.

Sinha, S., Nevett, C., Shuttleworth, C.A. and Kielty, C.M. (1998) Cellular and extracellular biology of the latent transforming growth factor-β binding proteins. Matrix Biol. 17:529–545.

Sporn, M.B. and Roberts, A.B. (1992) Transforming growth factor-β: recent progress and new challenges. Cell Biol. 119:1017–1021.

Stander, M., Naumann, U., Dumitrescu, L., Heneka, M., Loschmann, P., Gulbins, E., Dichgans, J. and Weller, M. (1998) Decorin gene transfer-mediated suppression of TGF-β synthesis abrogates experimental malignant glioma growth in vivo. Gene Ther. 5:1187–1194.

Suzu, S., Kimura, F., Matsumoto, H., Yamada, M., Hashimoto, K., Shimamura, S. and Motoyoshi, K. (1997) Identification of binding domains for basic fibroblast growth factor in proteoglycan macrophage colony-stimulating factor. Biochem. Biophys. Res. Commun. 230:392–397.

Suzu, S., Ohtsuki, T., Makishima, M., Yanai, N., Kawashima, T., Nagata, N. and Motoyoshi, K. (1992) Biological activity of a proteoglycan form of macrophage colony-stimulating factor and its binding to type V collagen. J. Biol. Chem. 267:16812–16815.

Suzu, S., Ohtsuki, T., Yanai, N., Takatsu, Z., Kawashima, T., Takaku, F., Nagata, N. and Motoyoshi, K. (1992) Identification of a high molecular weight macrophage colony-stimulating factor as a glycosaminoglycan-containing species. J. Biol. Chem. 267:4345–4348.

Svensson, L., Aszodi, A., Reinholt, F.P., Fässler, R., Heinegard, D. and Oldberg, A. (1999) Fibromodulin-null mice have abnormal collagen fibrils, tissue organization, and altered lumican deposition in tendon. J. Biol. Chem. 274:9636–9647.

Takeuchi, Y., Kodama, Y. and Matsumoto, T. (1994) Bone matrix decorin binds transforming growth factor-β and enhances its bioactivity. J. Biol. Chem. 269:32634–32638.

Turnbull, J.E. and Gallagher, J.T. (1993) Heparan sulphate: functional role as a modulator of fibroblast growth factor activity. Biochem. Soc. Trans. 21:477–482.

Vlodavsky, I., Miao, H.Q., Medalion, B., Danagher, P. and Ron, D. (1996) Involvement of heparan sulfate and related molecules in sequestration and growth promoting activity of fibroblast growth factor. Cancer Metastasis Rev. 15:177–186.

Wahl, S.M., Hunt, D.A., Wakefield, L.M., McCartney-Francis, N., Wahl, L.M., Roberts, A.B. and Sporn, M.B. (1987) Transforming growth factor type β induces monocyte chemotaxis and growth factor production. Proc. Natl. Acad. Sci. U S A 84:5788–5792.

Whinna, H.C., Choi, H.U., Rosenberg, L.C. and Church, F.C. (1993) Interaction of heparin cofactor II with biglycan and decorin. J. Biol. Chem. 268:3920–3924.

Winnemöller, M., Schmidt, G. and Kresse, H. (1991) Influence of decorin on fibroblast adhesion to fibronectin. Eur. J. Cell Biol. 54:10–17.

Xu, T., Bianco, P., Fisher, L.W., Longenecker, G., Smith, E., Goldstein, S., Bonadio, J., Boskey, A., Heegaard, A.M., Sommer, B., Satomura, K., Dominguez, P., Zhao, C., Kulkarni, A.B., Robey, P.G. and Young, M.F. (1998) Targeted disruption of the biglycan gene leads to an osteoporosis-like phenotype in mice. Nat. Genet. 20:78–82.

Yamaguchi, Y. and Ruoslahti, E. (1988) Expression of human proteoglycan in Chinese hamster ovary cells inhibits cell proliferation. Nature 336:244–246.

Yamaguchi, Y., Mann, D.M. and Ruoslahti, E. (1990) Negative regulation of transforming growth factor-β by the proteoglycan decorin. Nature 346:281–284.

Yayon, A., Klagsbrun, M., Esko, J.D., Leder, P. and Ornitz, D.M. (1991) Cell surface heparin molecules are required for binding of basic fibroblast growth factor to its high affinity receptor. Cell, 64: 841–848.

Zhang, J.D., Cousens, L.S., Barr, P.J. and Sprang, S.R. (1991) Three-dimensional structure of human basic fibroblast growth factor, a structural homolog ofinterleukin 1 β. Proc. Natl. Acad. Sci. U S A 88:3446–3450.

Zhang, Y., Cao, L., Yang, B.L. and Yang, B.B. (1998) The G3 domain of versican enhances cell proliferation via epidermial growth factor-like motifs. J. Biol. Chem. 273:21342–21351.

